# Heat stress-induced photosynthetic impairment and autophagy modulation in *Paris polyphylla* var. *yunnanensis*: A physiological and molecular Perspective

**DOI:** 10.1371/journal.pone.0354925

**Published:** 2026-07-31

**Authors:** Lixia Lin, Xueqin Zhang, Ruiyun Lai, Jianzhong Lin, Zanhua Zhong, Huihua Li

**Affiliations:** Fujian Institute of Subtropical Botany, Xiamen, Fujian, China; Nuclear Science and Technology Research Institute, IRAN, ISLAMIC REPUBLIC OF

## Abstract

High temperature is one of the major environmental stressors that severely affects plant growth. *Paris polyphylla* var. *yunnanensis*, a traditional Chinese herbal medicine, is sensitive to high temperature. However, the underlying mechanisms of its response to high temperature remain unclear. In this study, we investigated the physiological and proteomic change of *P. polyphylla* var. *yunnanensis* under different treatments (25°C, 30°C, 35°C, 40°C). Our results showed that high temperature directly impaired photosynthesis and disrupted metabolism, evidenced by reduced chlorophyll and photosynthetic rate, as well as accumulated proline and increased conductivity. A total of 893 differentially expressed proteins (DEPs) were identified, with significant changes in the expression levels of enzymes associated with protein processing and synthesis. Additionally, the expression levels of key proteins involved in the circadian pathway and the glutathione pathway were also notably upregulated. Dynamic changes in the endocytosis and autophagy-related proteins ATG3 and ATG8C were also observed, suggesting that these processes may play a significant protective role under high-temperature stress. Overall, this study provides an important starting point for improving the heat tolerance of *P.polyphylla* var. *yunnanensis* through genetic engineering.

## Introduction

As a traditional medicinal plant, *Paris polyphylla* var. *yunnanensis* is an important component of many Chinese medicines, including Yunnan Baiyao and Gongxuening [1]. *P. polyphylla* var. *yunnanensis* is highly sensitive to high-temperature stress, with an optimal growth temperature range of 15–25°C [2]. With the intensification of global warming, high-temperature stress has become a key environmental factor limiting both the distribution of wild *P. polyphylla* var. *yunnanensis* and the development of an industry devoted to its cultivation.

Global climate change has rendered elevated temperatures a critical environmental stressor impairing plant growth and agricultural productivity. High temperatures can shorten the growth cycle of crops, suppress photosynthetic efficiency, lead to leaf senescence, impair root function, and reduce water transport capacity, thereby significantly decreasing both the yield and quality of crops [3]. Specifically, hyperthermia disrupts mitotic progression, causes pollen sterility, reduces seed viability, and perturbs fruit morphogenesis [4]. Furthermore, elevated temperatures exacerbate edaphic water evaporation, creating pronounced xeric conditions in the rhizosphere that critically impair plant-water relations [5]. In response to these changes, plants mitigate heat absorption by modifying leaf orientation, curling their leaves, and closing stomata [6]. Additionally, they enhance thermal stability by increasing leaf thickness and reinforcing the structure of stems and leaves [7]. Plants also increase the content of osmotic protectants such as proline and soluble sugars to maintain cellular osmotic balance [8]. By upregulating the expression of antioxidant enzymes such as catalase (CAT), excess reactive oxygen species (ROS) can be effectively eliminated, thereby promoting the accumulation of antioxidants like glutathione (GSH) to safeguard against oxidative damage [9].

At the molecular level, plants activate multiple mechanisms, including heat stress transcription factors (HSFs), heat shock proteins (HSPs), and non-coding RNAs, to cope with high-temperature stress [10]. The integration of proteomics and bioinformatics has emerged as a powerful synergistic approach in the study of plant stress resistance, allowing for a comprehensive analysis of the complex mechanisms underlying stress responses and providing molecular insights into the physiological processes of plant adaptation [11]. Utilizing proteomics techniques to investigate the response mechanism of ginseng to high-temperature stress, this study found that the expression levels of heat shock proteins (HSPs) and antioxidant enzymes in ginseng leaves were significantly upregulated [12]. Wang et al. employed Tandem Mass Tag (TMT)-based quantitative proteomics, and reported that high-temperature stress induced 300 differentially expressed proteins (DEPs) in maca seedlings [13]. Similarly, Zhang et al. applied isobaric tags for relative and absolute quantification (iTRAQ) proteomics to demonstrate that high-temperature stress significantly inhibited expression of the rice granule-bound starch synthase (GBSS) gene as well as the starch branching enzyme (SBE) gene [14].

Although one previous study has found that high-temperature stress severely compromises the cell membrane stability of *P. polyphylla* var*. yunnanensis*, the molecular mechanism is still unclear [15]. Therefore, the present study aimed to systematically analyze the changes in protein expression patterns of *P. polyphylla* var. *yunnanensis* under high-temperature stress using proteomics techniques and to explore the molecular basis of its heat tolerance in combination with physiological and biochemical indicators.

## Materials and methods

### Plant materials

Three-year-old rhizomes with buds of *Paris polyphylla* var. *yunnanensis* (*Paris polyphylla* Smith var. *yunnanensis* (Franch.) Hand.-Mazz.), procured from Midu County, Dali Prefecture, Yunnan Province, China. All rhizomes were derived from the same vegetative propagation bath to ensure genetic uniformity. Rhizomes were transplanted into 18 cm × 14 cm culture pots (diameter × height) containing a 2:1 (v/v) mixture of humus and perlite (one rhizome per pot). Plants were acclimated for 60 days in a controlled greenhouse (20–25°C, 70–80% relative humidity, 75% shading, daily irrigation). Morphologically uniform, vigorously growing individuals showing no signs of pathogen infection or pest damage were selected for subsequent experiments. A total of 60 plants were used in this study. For each temperature treatment (25 °C, 30 °C, 35 °C, 40 °C and 45 °C), three biological replicates were performed, with each replicate consisting of four individual plants.

### Temperature treatment design

Seedlings of *P. polyphylla* var. *yunnanensis* were placed in a light incubator, (25°C, 75 ± 10% relative humidity, 400 µmol·m^-2^·s^-1^, 12 h:12 h light-dark photoperiod) and maintained for seven days with daily watering to maintain adequate soil moisture levels. To simulate progressive heat stress conditions, a stepwise temperature elevation regime was applied. Plants were assigned to five temperature treatments: 25°C (control), 30°C, 35°C, 40°C, and 45°C. For the control group, plants were maintained at 25°C for 48 h. For heat-treated groups, a gradual acclimation strategy was adopted to avoid abrupt thermal shock and to better reflect natural field temperature fluctuations. Specifically, plants in the 30°C group were transferred directly from 25°C to 30°C and maintained for 48 h. Plants in the 35°C group were first exposed to 30°C for 48 h and subsequently transferred to 35°C for another 48 h. Plants in the 40°C group were sequentially acclimated from 30°C to 35°C and finally to 40°C, with each step lasting 48 h. Similarly, plants in the 45°C group were sequentially acclimated from 30°C to 35°C, 40°C, and finally to 45°C, with each step lasting 48 h. At each stage, plants were maintained under identical light intensity, photoperiod, and humidity conditions to ensure that temperature was the only variable factor. All temperature increases were initiated at the start of the dark period. Upon completion of each respective temperature treatment, designated samples were flash-frozen in liquid nitrogen, and stored at −80°C. Each treatment included three biological replicates.

### Photosynthetic measurements

Net photosynthetic rate (P_n_) of the leaves of *P. polyphylla* var. *yunnanensis* was measured using a portable photosynthesis system (Li-6400, Li-Cor, Lincoln, NE, USA). Before application of high-temperature treatments, leaves with comparable P_n_ values were selected and marked for subsequent analysis. After the experiment commenced, measurement of photosynthetic parameters for *P. polyphylla* var. *yunnanensis* began on the second day during the light period (9:00 AM – 12:00 PM) following temperature adjustment. Parameters included P_n_, stomatal conductance (G_s_), intercellular CO_2_ concentration (C_i_), and transpiration rate (T_r_). Non-stomatal limitation index (C_i_/G_s_) and the stomatal limitation index (L_s_) were calculated using the formulas:


$$\begin{array}{c} {\text{L}}_{\text{s}}\;=\;\text{1}\;-\;({\text{C}}_{\text{i}}/{\text{C}}_{\text{a}}), \end{array}$$

where C_a_ was environmental CO_2_ concentration [16]. Across three plants, at least five leaves per treatment were analyzed (with greater than five replicates per leaf).

### Physiological and biochemical assays

Leaves were sampled from whole plants frozen after high-temperature exposure. Total chlorophyll content as well as the ratio of chlorophyll a to b were quantified using standard methods. Proline content was determined using the acidic ninhydrin reaction method [17]. Relative electrical conductivity (EL, %) was measured using a conductivity meter (Leici-DDS-307A, Shanghai, China) at room temperature for E1, and after boiling the blades for 20 minutes and subsequently cooling them to room temperature for E2. EL was calculated using the following formula:


$$\begin{array}{c} \text{Relative EL }(\%)\;=\;(\text{E1}/\text{E2})\;\times \text{ 100} \end{array}$$

Measurements followed manufacturer protocols (detection kits, Nanjing Jiancheng Bioengineering Institute, China).

### Protein extraction and trypsinization

Frozen leaf tissue (0.5 g) was ground in liquid nitrogen and suspended in four volumes of phenol extraction buffer (10 mM dithiothreitol, 1% protease inhibitor). Samples were subjected to ultrasonic lysis before an equal volume of tris-buffered phenol was added and samples were centrifuged (10 min, 5,500 g, 4°C). Overnight at 4°C, proteins were precipitated from the phenol phase in five volumes of cold 0.1 M ammonium acetate in methanol and pelleted by centrifugation. After discarding the supernatant, the pellet was washed twice with methanol, and once with acetone. Finally, the pellet was dissolved in 8 M urea. Protein concentrations were determined using a BCA assay kit.

For analysis, it was necessary to create subsamples of equal protein concentration from the samples. Equal protein amounts were adjusted to uniform volume using lysis buffer. Trichloroacetic acid (TCA, protein precipitant) was added to attain a final concentration of 20%, vortexed, incubated (4°C, 2 h), centrifuged (4500 g, 5 min, 4°C), and the pellet washed 3 times with cold acetone. Proteins were redissolved in 200 mM TEAB buffer with ultrasound. Digestion was performed overnight with trypsin at a ratio of 1:50 (trypsin:protein, m/m) at 37°C. Dithiothreitol was added to achieve a final concentration of 5 mM, followed by incubation (56°C, 30 min). Iodoacetamide was then added to reach a final concentration of 11 mM, and the mixture was incubated for 15 minutes at room temperature in the dark.

### LC-MS/MS analysis

Peptides were dissolved in liquid chromatography mobile phase A (aqueous, 0.1% formic acid, 2% acetonitrile) and separated using an NanoElute ultra-high-performance liquid chromatography (UHPLC) system. Mobile phase A and mobile phase B (aqueous, 0.1% formic acid, 90% acetonitrile) were used to elute the digested peptides. The liquid phase gradient settings were: 0–9 min, 6–24% B; 9–11 min, 24%−35% B; 11–13 min, 35%−80% B; 13–15 min, 80% B and the flow rate kept at 500 nL/min. After separation, isolated peptides were analyzed by timsTOF Pro mass spectrometer (Bruker Daltonics), with capillary ionization (1.75 kV). The data acquisition mode utilized in this study was data-independent parallel accumulation-serial fragmentation (dia-PASEF). The first-level mass spectrometry scan was conducted over a range of 300–1500 m/z. Following the acquisition of one first-level mass spectrum, 20 PASEF mode acquisitions were executed. The secondary mass spectrometry scan occurred within a range of 400–850 m/z, employing a window of 7 m/z intervals.

### Bioinformatics analysis

Mass spectra were searched against a protein database using DIA-NN v1.8. The protein database was constructed from the *P. polyphylla* var. *yunnanensis* full-length transcriptome, which was generated from equal amounts of pooled leaf samples of the 25°C, 30°C, 35°C, and 40°C treatment groups using PacBio UMI ISO-Seq (BioProject PRJNA1467211). Parameters: enzyme Trypsin/P with one missed cleavage, fixed carbamidomethylation (+57.021 Da), and N-terminal methionine removal. Mass accuracy was set to 10 ppm, with peptide/protein FDR < 0.01. Quality control required >5000 peptides identified and consistent retention times. Differential protein expression was assessed using Student’s t-test (|FC| > 1.5, P < 0.05). Functional annotation included Gene Ontology (GO) and Kyoto Encyclopedia of Genes and Genomes (KEGG) functional enrichment analyses. Protein interaction networks were constructed utilizing STRING and Cytoscape. The results were visualized, with volcano plots and principal component analysis (PCA).

### Differential protein screening

Mass spectra were processed using DIA-NN software, for peak recognition, matching, and protein identification based on species-specific databases. Subsequently, the identified protein abundance data were subjected to normalization using the limma package in R. This process was implemented to correct for systematic errors. A total of 6,355 proteins were identified and quantified across all samples, serving as the background dataset for differential expression analysis. Differentially expressed proteins (DEPs) meeting the above criteria were selected for downstream pathway and functional analyses.

### qRT-PCR verification of gene expression of candidate proteins

RT-qPCR was employed to validate the six key genes identified by proteomic analysis. Total RNA was extracted from three replicate plants per treatment using TRIzol™ reagent (Invitrogen), and cDNA was synthesized from 1 μg of total RNA using the Jumbo™ Script Enzyme Mix. Each 20 μL reaction mixture contained 10 μL of 2 × ChamQ Universal SYBR qPCR Master Mix, 2 μL of cDNA, and 0.4 μL of each primer (10 μM). The PCR program consisted of one cycle at 95°C for 30 s, followed by 40 cycles of 95°C for 10 s and 60°C for 30 s. Relative expression levels were calculated using the 2^(-ΔΔCt) method. Three technical replicates were performed for each sample. The corresponding mRNA sequences were obtained by querying the identified protein sequences against the *P. polyphylla* var. *yunnanensis* full-length transcriptome database (PRJNA1467211) using tBLASTn. Primers were designed using Primer Premier 5 (Premier Biosoft Inc., CA). The primers used for RT-qPCR are listed in S1 Table.

### Statistic analysis

Data were processed using Microsoft Excel 2020 (Microsoft Inc., USA) and IBM SPSS Statistics (version 25.0). Graphs were generated using GraphPad Prism 8.0 (GraphPad Software, USA). Results are expressed as the mean ± standard error (SE) or proportion ± SE. One-way analysis of variance (ANOVA) followed by Duncan’s multiple range test was used for comparisons, with significance accepted at equal to or less than 0.05.

## Results

### Morphological changes of *P. polyphylla* var. *yunnanensis* under high temperature stress

**Fig 1:**
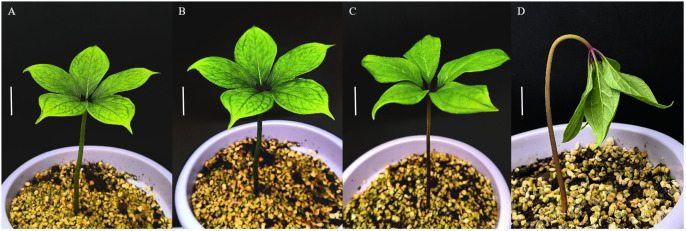
Morphological changes in *P. polyphylla* var. *yunnanensis* under different temperature treatments. (A) 25°C for 48 h (Control). (B) 30°C for 48 h. (C) 35°C for 48 h. (D) 40°C for 48 h. Scale bar = 5 cm.

Temperature treatments induced distinct morphological alterations in *P. polyphylla* var. *yunnanensis* Compared to control plants (25°C, Fig 1A), those subjected to 30°C (Fig 1B)maintained healthy foliage with no visible phenotypic abnormalities. At 35°C (Fig 1C), leaf tips and margins exhibited downward curling, while 40°C(Fig 1D) triggered severe structural collapse and pronounced leaf curling. Plants exposed to 45°C exhibited rapid wilting and complete mortality within 48 h (n = 12), precluding further physiological, photosynthetic, and proteomic analysis.

### Changes of photosynthetic characteristics in response to heat stress

**Fig 2:**
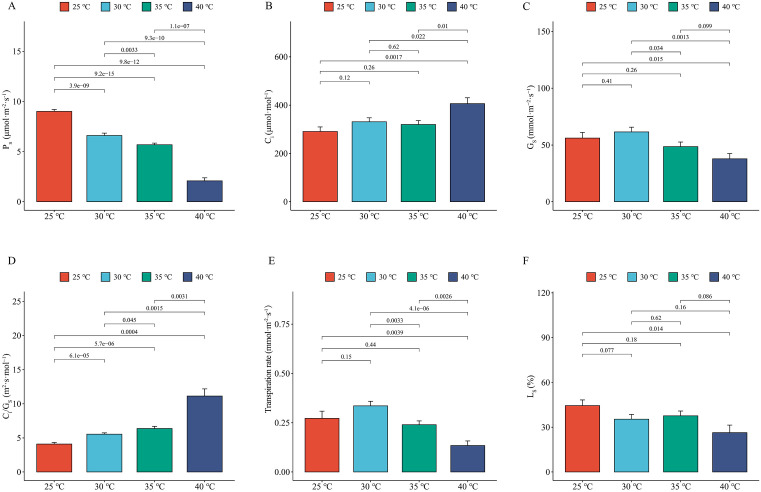
Photosynthetic characteristics in the leaves of *P. polyphylla* var. *yunnanensis* under heat stress. (A) Net photosynthetic rate (P_n_). (B) Intercellular CO_2_ concentration (C_i_). (C) Stomatal conductance (G_S_). (D) Ratio of C_i_/G_S_. (E) Transpiration rate. (F) Stomatal limitation value (L_S_). Statistical significance between treatments is indicated above bars.

As shown in Fig 2, increasing temperature resulted in alterations to the photosynthetic parameters of both the control group and the high-temperature group of *P. polyphylla* var. *yunnanensis* leaves. The net photosynthetic rate (P_n_) declined significantly, with decreases of 27.07%, 37.14%, and 76.62% at 30°C, 35°C, and 40°C, respectively, compared with the control (Fig 2A). Intercellular CO_2_ concentration (C_i_) and the C_i_/G_S_ ratio generally increased. C_i_ rose by 14.5%, 9.6%, and 39.0% at 30°C, 35°C, and 40°C, respectively (Fig 2B). The C_i_/G_S_ ratio peaked at 40°C, suggesting that the limiting factor shifted from stomatal to non-stomatal processes. Stomatal conductance (G_S_) and transpiration rate (T_r_) initially increased and then decreased. Both peaked at 30°C, then declined by 11.7% and 30.8% (G_S_), and by 15.5% and 52.7% (T_r_) at 35°C and 40°C, respectively (Fig 2C, 2E). The stomatal limitation value (L_s_) decreased by 20.44%, 15.33%, and 40.68% at 30°C, 35°C, and 40°C compared with the control (Fig 2F).

### Physiological responses to heat stress Heat stress

**Fig 3:**
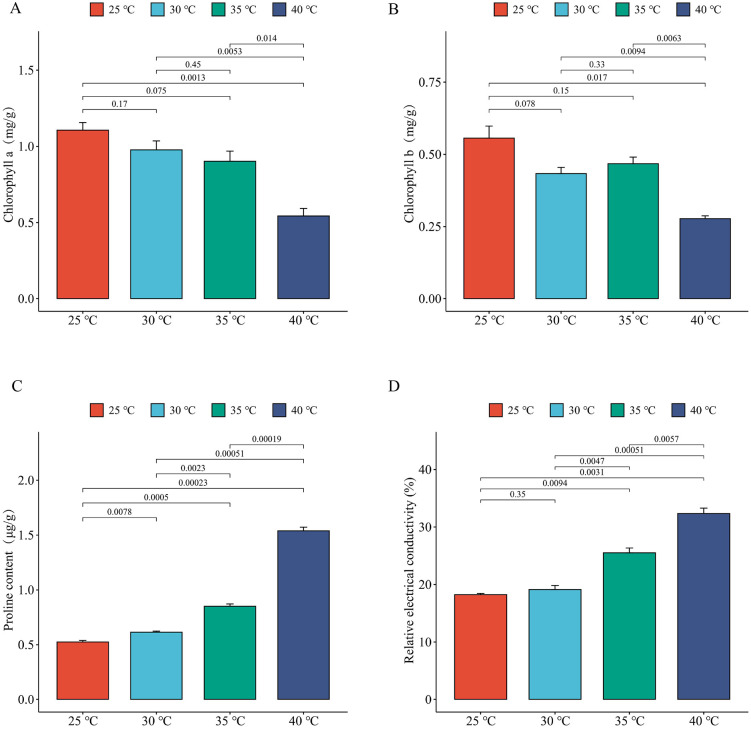
The physiological characteristics of *P. polyphylla* var. *yunnanensis* under heat stress. (A) Chlorophyll a content (mg/g). (B) Chlorophyll b content (mg/g). (C) Proline content (μg/g). (D) Relative electrolyte conductivity (%). Statistical significance between treatments is indicated above bars.

Chlorophyll a and b both declined with rising temperature. Chlorophyll a declined by 23%, 34%, and 12% at 30°C, 35°C, and 40°C, respectively, compared with 25°C (Fig 3A). Chlorophyll b decreased by 22.17%, 19.60%, and 50.15% at the same temperatures (Fig 3B). Under heat stress, the proline content exhibited a significant increasing trend, rising by 16.95%, 62.10%, and 193.14% at 30°C, 35°C, and 40°C, respectively (Fig 3C). As shown in Fig 3D, relative electrolyte conductivity increased with temperature, with no significant difference at 30°C, but increases of 21.50% and 25.35% at 35°C and 40°C, respectively.

### Repeatability analysis

**Fig 4:**
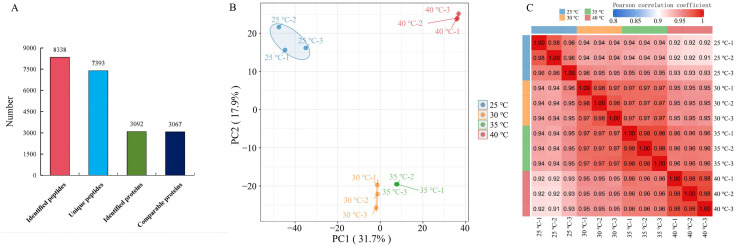
DIA-based proteomic analysis of *P. polyphylla* var. *yunnanensis* under different temperature treatments. (A) Summary of MS/MS spectra and identified differentially expressed proteins (DEPs). (B) Principal component analysis of samples from different temperature treatment groups. (C) Pearson correlation coefficients for three biological replicates within each treatment.

Proteomic analysis (LC-MS/MS, see methods), yielded a total of 8,338 identified peptides and 7,393 unique peptides. After annotation, 3092 proteins were identified, and 3067 comparable proteins were obtained, respectively (Fig 4A).

Principal component analysis (PCA) based on the relative quantification values of all samples, showed that biological replicates clustered tightly within groups and separated clearly between treatments (Fig 4B). Pearson correlation coefficients between replicates exceeded 0.91, with several approaching 1 (Fig 4C). This confirms the strong reproducibility of the experimental data and its downstream analysis.

### Differentially expressed proteins

**Fig 5:**
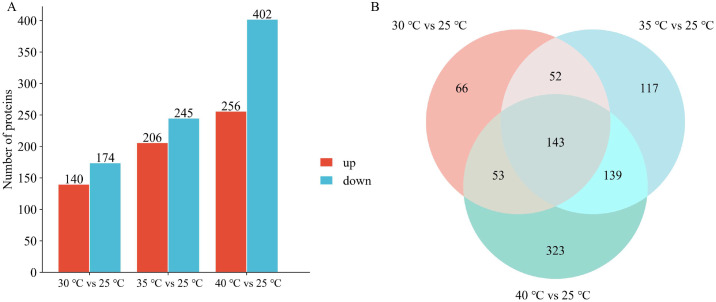
Analysis of differentially expressed proteins (DEPs). (A) Number of up-regulated and down-regulated DEPs in different comparisons. (B) Venn diagram of DEPs among different comparison groups.

In this study, we compared different temperature treatments of differentially expressed proteins (DEPs, defined in section 2.8). Significant DEPs were obtained from the comparisons of samples at 30°C, 35°C and 40°C (Fig 5A). Compared with the control, 314 DEPs (140 upregulated, 174 downregulated) were found at 30°C; 451 (206 up, 245 down) at 35°C; and 658 (256 up, 402 down) at 40°C.

The number of upregulated DEPs at 35°C was 1.47-fold that at 30°C, and the number of downregulated DEPs was 1.41-fold greater. At 40°C, upregulated DEPs increased 1.24-fold and downregulated DEPs 1.64-fold compared with 35°C.

Pairwise comparisons identified 195 and 196 DEPs between 30°C vs 35°C and 30°C vs 40°C, respectively, and 282 between 35°C and 40°C (Fig 5B). Across comparisons, 143 DEPs were common. These results indicate that *P. polyphylla* var. *yunnanensis* responds to increasing heat stress by both inducing and repressing protein synthesis.

### KEGG analysis

**Fig 6:**
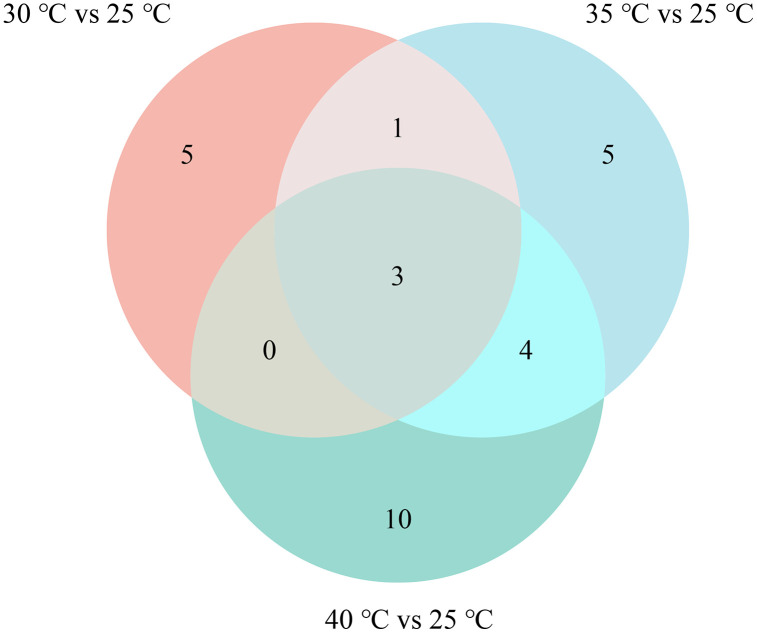
Venn diagram showing the overlap of KEGG-enriched pathways among different temperature treatment comparisons.

Venn analysis of KEGG pathways among the three comparison groups (P < 0.05), showed overlap across comparisons, including metabolic pathways, biosynthesis of secondary metabolites, and porphyrin metabolism (Fig 6).

**Fig 7:**
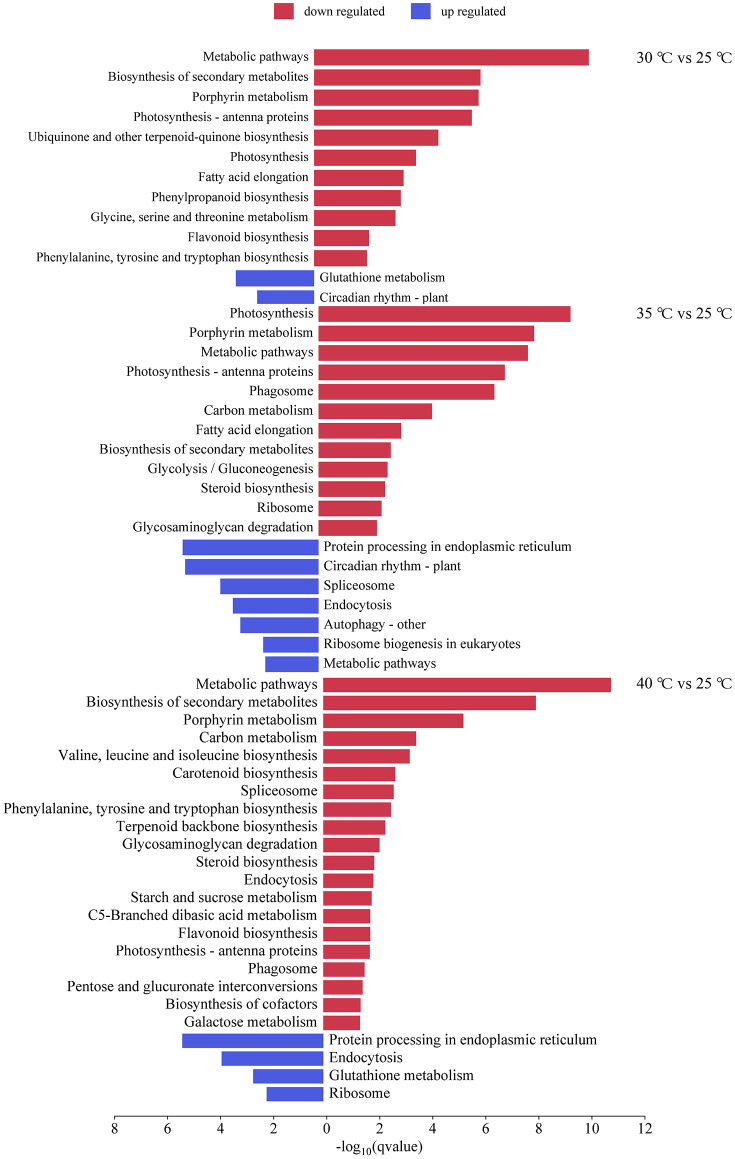
KEGG pathway enrichment analysis showing significantly enriched pathways in different temperature treatment comparisons.

At 30°C, 13 pathways were significantly enriched, including upregulation of glutathione metabolism and circadian rhythm–plant, with 11 downregulated. At 35°C, 19 pathways were enriched, seven upregulated (protein processing in the endoplasmic reticulum, circadian rhythm-plant, spliceosome, endocytosis, autophagy-other, ribosome biogenesis in eukaryotes, and metabolic pathways,) and 12 downregulated. At 40°C, 24 pathways were enriched, four upregulated (protein processing in the endoplasmic reticulum, endocytosis, glutathione metabolism, and ribosome,) and 20 downregulated (Fig 7).

At 30°C, *P. polyphylla* var. *yunnanensis* rapidly activated the glutathione (GSH) metabolic pathway to remove reactive oxygen species (ROS) and promote the maintenance of cellular redox homeostasis. At 35°C, although heat stress persists, the accumulation of reactive oxygen species (ROS) was insufficient to induce a significant enrichment of the glutathione metabolic pathway, thus no statistical differences were observed in the KEGG functional enrichment analysis. At 40°C, damage to the membrane system was induced, causing protein denaturation, and an excessive accumulation of ROS, activating the glutathione metabolic pathway to eliminate excess ROS and repair oxidative damage.

### Heat map analysis

**Fig 8:**
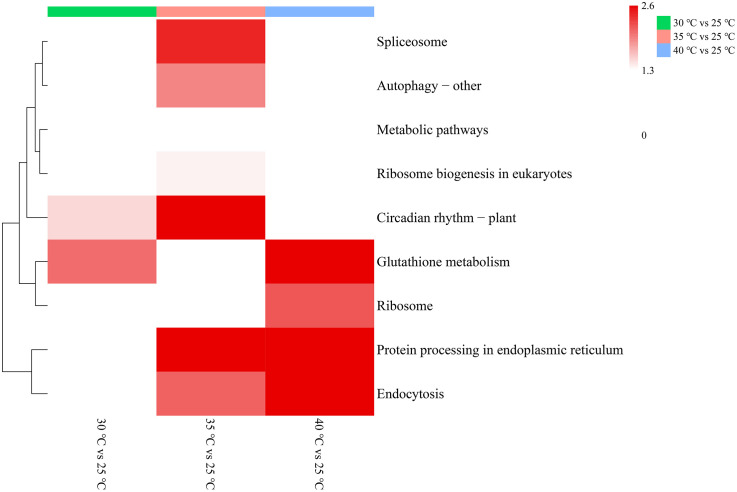
Heat map of KEGG pathway enrichment for up-regulated proteins among different comparison groups.

Heatmap results (Fig 8) indicated that the number of upregulated KEGG pathways increased at 35°C and subsequently declined at 40°C. At 30°C, only the expression levels of the glutathione metabolism and circadian rhythm-plant KEGG pathways were significantly upregulated. Seven KEGG pathways showed significant upregulation at 35°C. Notably, the pathways associated with endoplasmic reticulum protein processing, spliceosome activity, endocytosis, and autophagy, suggesting an enhancement of protein homeostasis and stress response mechanisms at this temperature. Additionally, the circadian rhythm-plant KEGG pathway exhibited the highest induction effect at 35°C. At 40°C, there was a significant upregulation of endoplasmic reticulum protein processing, endocytosis, glutathione metabolism, and ribosome activity. This observation reflected an enhancement of the endoplasmic reticulum stress response and an increase in protein turnover. It was evident that 35°C can induce a variety of adaptive responses, whereas extreme temperature 40°C predominantly activated protein repair and degradation mechanisms.

**Fig 9:**
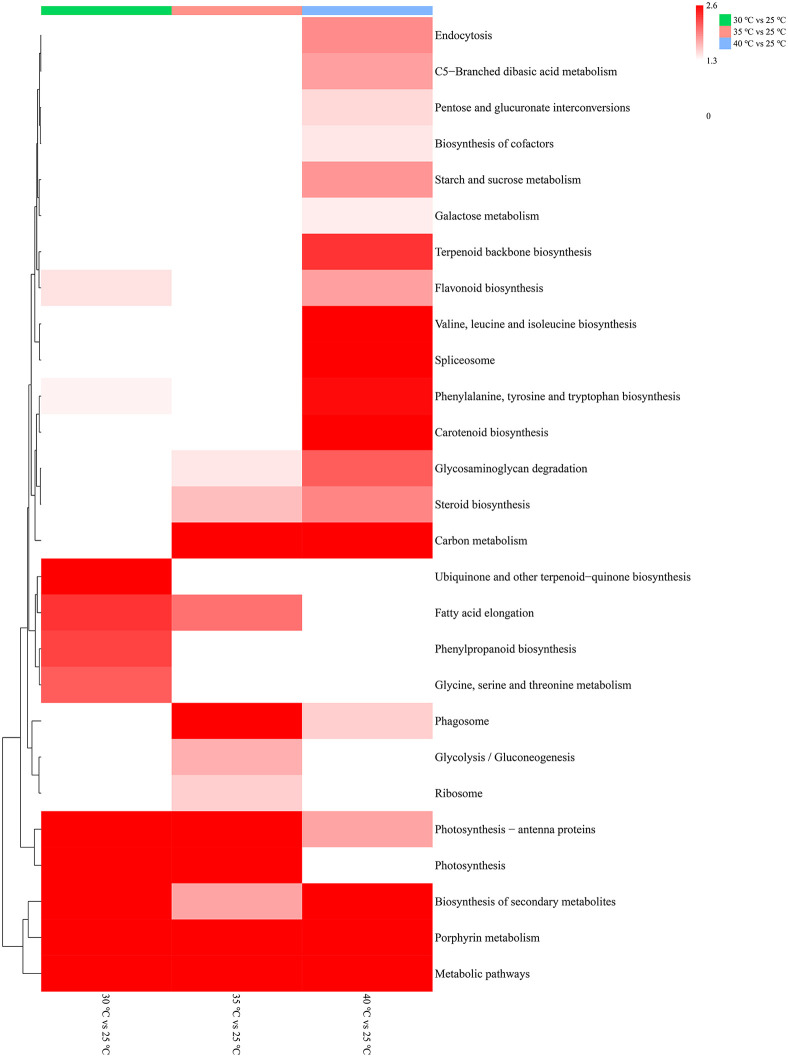
Heat map of KEGG pathway enrichment for down-regulated proteins among different comparison groups.

Downregulation of related pathways increased at higher temperatures (30°C, 35°C, and 40°C) (Fig 9). This observation is consistent with previous findings regarding the increase in downregulated proteins. At 30°C, fatty acid elongation, ubiquinone and other terpenoid-quinone biosynthesis, photosynthetic-antenna proteins, porphyrin metabolism, and phenylpropanoid biosynthesis pathways were downregulated, indicating early impacts on lipid metabolism, photosynthetic efficiency, and secondary metabolim. At 35°C, photosynthesis, photosynthesis – antenna proteins, and porphyrin metabolism exhibited the most significant downregulation, indicating a severe impairment of light-dependent energy metabolism. Furthermore, both phagosome and steroid biosynthesis were notably affected, underscoring the potential impact on cellular defense and membrane stability. At 40°C the biosynthesis of secondary metabolites, and carbon metabolism, were strongly downregulated indicating a substantial disruption of central metabolism and biosynthetic processes. It was noteworthy that the biosynthesis of valine, leucine, and isoleucine, along with carotenoids and flavonoids, was also significantly downregulated. This suggested that the capacity for stress recovery and antioxidant production was compromised. These findings indicated that a temperature of 35°C primarily affected pathways associated with photosynthesis, whereas a temperature of 40°C induced systemic dysfunctions in both primary and secondary metabolism.

### Functional classification of identified proteins

Proteins responsive to heat stress were functionally classified into upregulated (Table 1) and downregulated groups (Table 2). In the photosynthesis pathway (map00195) (Table 2), core components of photosystem I and II were significantly downregulated, including subunits of Photosystem I. Specifically psaK (isoform_10551), psaN (isoform_105195), and psaXI (isoform_35860), demonstrated the most pronounced downregulation at 35°C. Additionally, key components of Photosystem II, including CP43 (isoform_10549), CP47 (isoform_135638), D1 protein (isoform_192912), and D2 protein (isoform_139045), also showed temperature-dependent downregulation, with the D1 protein exhibiting the most significant reduction at 35°C. The cytochrome b6-f complex (isoform_119127, isoform_168481) and ferredoxin-NADP+ reductase (isoform_103844) also exhibited a trend of downregulation. These changes suggested that high-temperature stress significantly inhibits the expression of proteins related to photosynthesis.

**Table 1:** The information of up regulated DEPs during heat acclimation process.

Protein accession	Protein description	Coverage (%)	Peptides number	Subcellular localization	Log2(Fold change)		
**30**°C **vs 25**°C	**35**°C **vs 25**°C	**40**°C **vs 25**°C
*map03040 Spliceosome*							
*isoform_113471*	Splicing factor U2af small subunit B	6.59	2	nucleus	0.34	0.68	0.72
*isoform_103306*	Peptidyl-prolyl cis-trans isomerase CYP22	8.52	2	chloroplast	0.26	0.63	0.14
*isoform_136850*	Serine/arginine-rich splicing factor SR30	13.70	2	cytoplasm	0.62	0.59	1.56
*isoform_230457*	Heat shock 70 kDa protein	13.74	8	cytoplasm	0.2	0.97	1.33
*isoform_225907*	Heat shock 70 kDa protein 4	35.33	21	cytoplasm	0.63	0.89	1.01
*isoform_94508*	Heat shock cognate 70 kDa protein	9.09	6	chloroplast	0.58	0.95	1.03
*isoform_63193*	Heat shock cognate 70 kDa protein 2	29.42	18	cytoplasm	0.47	0.78	0.94
*isoform_68052*	Heat shock cognate 70 kDa protein 2	29.70	18	cytoplasm	0.14	0.35	0.65
*map03010 Ribosome*							
*isoform_85894*	40S ribosomal protein S4-3	16.33	4	cytoplasm	1.85	1.08	−0.89
*isoform_124694*	40S ribosomal protein S6	15.14	5	nucleus	0.07	0.62	−0.03
*isoform_105123*	60S ribosomal protein L7a-1	19.13	5	chloroplast	0.49	0.62	0.65
*isoform_10207*	60S ribosomal protein L19-3	14.91	2	nucleus	−0.05	0.46	0.80
*isoform_162745*	60S ribosomal protein L27-2	10.06	2	chloroplast	−0.11	0.35	0.65
*map04141 Protein processing in endoplasmic reticulum*							
*isoform_60212*	Chaperone protein dnaJ A6	6.84	3	nucleus	0.69	0.70	1.40
*isoform_137195*	DnaJ protein homolog	18.51	10	nucleus	0.25	0.37	0.85
*isoform_100608*	Ubiquitin-conjugating enzyme E2 28	7.43	1	nucleus	0.24	0.70	0.36
*isoform_11379*	SKP1-like protein 1	18.75	5	cytoplasm	0.45	0.75	0.97
*isoform_65297*	Cell division cycle protein 48 homolog	19.70	17	chloroplast	0.25	0.43	0.63
*isoform_166515*	Cell division cycle protein 48 homolog	18.30	8	cytoplasm	0.25	0.47	0.84
*isoform_176797*	16.9 kDa class I heat shock protein 1	11.98	2	cytoplasm	0.90	1.17	1.35
*isoform_101392*	18.2 kDa class I heat shock protein	36.48	6	cytoplasm	0.28	1.16	1.16
*isoform_230457*	Heat shock 70 kDa protein	13.74	8	cytoplasm	0.20	0.97	1.33
*isoform_225907*	Heat shock 70 kDa protein 4	35.33	21	cytoplasm	0.63	0.89	1.01
*isoform_94508*	Heat shock cognate 70 kDa protein	9.09	6	chloroplast	0.58	0.95	1.03
*isoform_63193*	Heat shock cognate 70 kDa protein 2	29.42	18	cytoplasm	0.47	0.78	0.94
*isoform_68052*	Heat shock cognate 70 kDa protein 2	29.70	18	cytoplasm	0.14	0.35	0.65
*map04712 Circadian rhythm – plant*							
*isoform_183037*	Casein kinase II subunit alpha-2	9.77	3	chloroplast	0.36	0.60	0.91
*isoform_121679*	Cryptochrome-1	8.37	5	mitochondria	0.64	0.62	0.11
*isoform_106873*	Protein GIGANTEA	3.47	2	nucleus	0.11	0.71	0.45
*isoform_102627*	Transcription factor HY5	7.19	1	nucleus	0.92	1.26	1.36
*map00480 Glutathione metabolism*							
*isoform_10018*	Glutathione S-transferase U18	4.48	1	cytoplasm	1.26	1.28	2.47
*isoform_100561*	Glutathione S-transferase U18	4.46	1	cytoplasm	0.51	0.57	0.79
*isoform_120194*	L-ascorbate peroxidase, cytosolic	12.73	4	mitochondria	0.48	0.62	0.92
*isoform_259819*	Peroxisomal isocitrate dehydrogenase [NADP]	20.34	3	cytoplasm	0.67	0.75	0.93
*map00380 Tryptophan metabolism*							
*isoform_133789*	Catalase isozyme 1	10.57	5	cytoplasm	0.50	0.55	0.79
*map04144 Endocytosis*							
*isoform_104391*	Ras-related protein Rab7	32.20	7	chloroplast	0.44	0.45	0.72
*isoform_175912*	Ras-related protein Rab7	14.56	3	chloroplast	0.25	0.74	1.57
*isoform_117175*	Vacuolar protein sorting-associated protein 26A	9.68	2	cytoplasm	0.48	0.56	0.65
*isoform_134178*	Vacuolar protein sorting-associated protein 28 homolog 1	6.16	1	chloroplast	0.40	0.51	0.95
*isoform_143835*	Vacuolar protein sorting-associated protein 32 homolog 1	25.69	5	chloroplast	0.43	0.68	0.53
*isoform_102382*	ADP-ribosylation factor 1	15.46	3	cytoplasm	0.21	0.28	0.63
*isoform_230457*	Heat shock 70 kDa protein	13.74	8	cytoplasm	0.20	0.97	1.33
*isoform_225907*	Heat shock 70 kDa protein 4	35.33	21	cytoplasm	0.63	0.89	1.01
*isoform_94508*	Heat shock cognate 70 kDa protein	9.09	6	chloroplast	0.58	0.95	1.03
*isoform_63193*	Heat shock cognate 70 kDa protein 2	29.42	18	cytoplasm	0.47	0.78	0.94
*isoform_68052*	Heat shock cognate 70 kDa protein 2	29.70	18	cytoplasm	0.14	0.35	0.65
*map04136 Autophagy – other*							
*isoform_116220*	Autophagy-related protein 3	22.15	3	cytoplasm	0.63	0.75	0.90
*isoform_11267*	Autophagy-related protein 8C	13.01	2	cytoplasm	0.53	0.63	1.09

**Table 2:** The information of down regulated DEPs during heat acclimation process.

Protein accession	Protein description	Coverage (%)	Peptides number	Subcellular localization	Log2(Fold change)		
					30°C vs 25°C	35°C vs 25°C	40°C vs 25°C
map00195 Photosynthesis							
*isoform_10551*	Photosystem I reaction center subunit psaK, chloroplastic	6.87	1	chloroplast	−0.28	−0.32	0.05
*isoform_105195*	Photosystem I reaction center subunit N, chloroplastic	10.59	2	chloroplast	−0.55	−0.60	−0.27
*isoform_35860*	Photosystem I reaction center subunit XI, chloroplastic	7.55	1	chloroplast	−0.36	−0.40	−0.02
*isoform_100012*	Photosystem I reaction center subunit XI, chloroplastic	3.64	1	chloroplast	−0.25	−0.29	−0.03
*isoform_10549*	Photosystem II CP43 reaction center protein	35.76	6	chloroplast	−0.05	−0.10	−0.18
*isoform_135638*	Photosystem II CP47 reaction center protein	12.71	7	plasma membrane	−0.25	−0.26	−0.22
*isoform_192912*	Photosystem II protein D1	24.25	7	plasma membrane	−0.62	−0.64	−0.40
*isoform_223992*	Photosystem II protein D1	26.35	9	plasma membrane	−0.15	−0.18	−0.28
*isoform_139045*	Photosystem II D2 protein	21.93	4	chloroplast	−0.10	−0.14	−0.18
*isoform_102184*	Photosystem II 10 kDa polypeptide, chloroplastic	6.62	2	plasma membrane	−0.38	−0.37	−0.36
*isoform_119127*	Cytochrome b6	12.5	3	chloroplast	−0.12	−0.20	−0.08
*isoform_125333*	Cytochrome b6-f complex subunit 4	6.25	1	plasma membrane	−0.16	−0.19	0.00
*isoform_168481*	Cytochrome f	21.71	7	chloroplast	−0.13	−0.18	−0.08
*isoform_103844*	Ferredoxin--NADP reductase, leaf isozyme 1, chloroplastic	21.56	5	cytoplasm	−0.12	−0.28	−0.20
*isoform_69738*	Ferredoxin--NADP reductase, leaf-type isozyme, chloroplastic	19.07	7	chloroplast	−0.08	−0.19	−0.10

In the spliceosome pathway (Table 1), the U2 auxiliary factor small subunit B splicing factor (isoform_113471) and splicing factor SR30 (isoform_136850) were both upregulated. Several members of the heat shock protein family were also upregulated with heat stress, including 70 kDa heat shock proteins (isoform_230457 and isoform_225907) (Table 1) and their homologs (isoform_94508, isoform_63193, and isoform_68052) (Table 1), along with two small heat shock proteins, 16.9 sHSP (isoform_176797) (Table 1) and 18.2 sHSP (isoform_101392) (Table 1). The 40S ribosomal protein S4-3 (isoform_85894) (Table 1), the 60S ribosomal protein L19-3 (isoform_10207) (Table 1), and the L27-2 protein (isoform_162745) (Table 1) exhibited significant upregulation at 35°C. The transcription factor HY5 (isoform_102627) (Table 1), which was involved in the plant circadian rhythm pathway, and the glutathione S-transferase U18 (isoform_10018) (Table 1), associated with the glutathione metabolism pathway, were significantly upregulated at 40°C. The expression levels of Rab7 protein (isoform_104391, isoform_175912) (Table 1), which were involved in endocytosis, as well as ATG3 (isoform_116220) (Table 1) and ATG8C (isoform_11267) (Table 1) in the autophagy pathway, gradually increased with rising temperatures.

### Verification of gene expression by RT-qPCR

**Fig 10:**
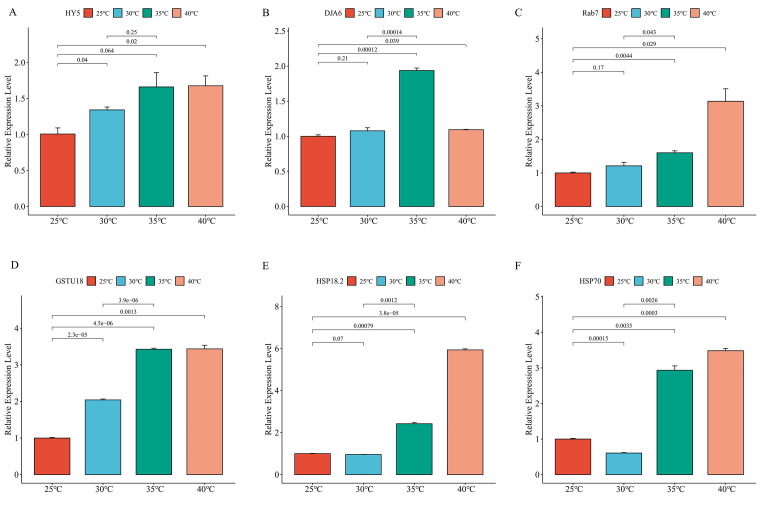
Validation of gene expression by RT-qPCR in *P. polyphylla* var. *yunnanensis* under heat stress. (A) *HY5*. (B) *DJA6*. (C) *Rab7*. (D) *GSTU18*. (E) *HSP18.2*. (F) *HSP70*. Statistical significance between treatments is indicated above bars.

RT-qPCR was performed to validate the reliability of the proteomic data. Among the six selected genes (*HY5*, *DJA6*, *Rab7*, *GSTU18*, *HSP18.2*, *HSP70*), most showed upregulation at higher temperatures, consistent with the proteomic findings (Fig 10). However, the correlation between protein and mRNA expression levels varied among genes and temperature conditions. Overall, the gene expression patterns were largely in agreement with the DIA-based proteomic data.

## Discussion

### High temperature stress affects photosynthesis

Based on both morphological and physiological evidence, 35°C represents a critical threshold for heat stress in *P. polyphylla* var. *yunnanensis* under our experimental conditions. It should be noted that this threshold was determined under constant‑temperature exposure for a fixed duration. In natural environments, factors such as exposure time, humidity, and temperature fluctuations may influence the actual threshold at which irreversible damage occurs. Therefore, plants treated at 35°C were selected as experimental material for detailed investigation of heat-stress response mechanisms. Photosynthetic indices revealed that net photosynthetic rate (P_n_) decreased by 27.01% at 30°C compared with 25°C, by 15.06% at 35°C compared with 30°C, and by 68.12% at 40°C compared with 35°C. The C_i_/G_s_ ratio increased by 59.8% at 30°C compared with 25°C, by 12.0% at 35°C compared with 30°C, and by 89.9% at 40°C compared with 35°C. These patterns suggest that non-stomatal factors are the primary limitation on photosynthesis as temperature increases. The most likely cause is the degradation of intracellular proteins or enzymes essential for photosynthesis.

High-temperature stress significantly disrupted photosynthesis by impairing photosystem I (PSI) and photosystem II (PSII) function. Proteins involved in photosynthetic processes were differentially regulated under heat stress [18]. As key PSI components, PsaK, PsaN, and PsaXI regulate light harvesting and electron transfer, and their abundance directly influences photosynthetic capacity [19]. For instance, overexpression of PsaK and PsaN in the rice mutant C3H69-OE-Oz-0 enhanced photosynthesis compared with wild type [20], while PSI-N mutants in Arabidopsis thaliana exhibited reduced photosynthesis, chlorophyll content, and growth [21]. Consistent with these findings, our proteomic data show that PsaXI, PsaN, and PsaK were consistently downregulated under heat stress, with the lowest abundance detected at 35°C. These results suggest that inhibition of PSI protein synthesis contributes to the decline in photosynthesis under high-temperature stress.

In PSII, the electron transfer chain and photochemical reactions were also disrupted, consistent with the thermosensitivity of this photosystem [22]. Core proteins of the PSII reaction center D1, D2, CP43, and CP47 play essential roles in maintaining electron transfer and stability [23]. Heat-induced electron leakage can damage the D1–D2 complex [24], while phosphorylation of CP43 stabilizes PSII dimers and delays D1 degradation [25]. CP47 ensures efficient excitation energy transfer [26]. Previous studies have shown that heat stress decreases PsbB (CP47) and PsbC (CP43) protein abundance in Chlamydomonas reinhardtii [27, 28]. In our study, D1 (isoform_192912), D2 (isoform_223992), CP43 (isoform_223992), and CP47 (isoform_135638) were all significantly downregulated, particularly at 35°C, where D1 abundance was lowest. These findings suggest that heat stress directly damages PSII core proteins, inhibits repair pathways, and suppresses the synthesis of D1, D2, CP43, and CP47, thereby reducing light capture and electron transfer efficiency.The cytochrome b6f complex, which transfers electrons within PSII, was also inhibited under high temperature [29,30]. In rice (Oryza meridionalis), both abundance and expression of ferredoxin-NADP+ reductase decrease after 24 h of heat exposure [31]. Consistent with this, we observed downregulation of both the cytochrome b6f complex and ferredoxin-NADP+ reductase at 35°C. These results suggest that the photosynthetic electron transport chain (PETC) of *P. polyphylla* var. *yunnanensis* is systematically impaired under heat stress.

### High temperature affects RNA processing and protein synthesis

Numerous studies have shown that heat stress can affect the differential expression of splicing factors and alter splicing patterns [32]. The spliceosome is composed of five small nuclear ribonucleoprotein complexes (snRNPs): U1, U2, U4, U5, and U6, along with hundreds of associated proteins [33]. Liu et al. found that the expression levels of the core components of the spliceosome (U2 snRNP) in Rhododendron moulmainense increased following high-temperature stress [34]. SR30 is a splicing factor enriched in serine and arginine residues, which plays a crucial role in spliceosome assembly and the regulation of specific gene splicing in plants [35]. Zhang et al. found that the SR30 gene in Arabidopsis thaliana was significantly upregulated under heat stress, indicating that SR30 may play a crucial role in the plant’s heat response by regulating splicing events [36]. Peptidyl-prolyl cis-trans isomerases (CYPs) maintain protein stability and reduce the accumulation of ROS by catalyzing the cis-trans isomerization of proteins, thereby protecting cells from oxidative damage [37]. Jo Seung Hee et al. discovered that CYP18−1 participates in RNA processing under heat stress in Arabidopsis by promoting the dephosphorylation of PRP18 and the splicing of introns [38]. In our research, we discovered that the splicing factor U2AF small subunit B, SR30, and CYP22 proteins were all highly expressed under high-temperature stress. Notably, under the 35°C treatment, the expression level of CYP22 was the highest compared to other treatments. Therefore, it is speculated that under high-temperature stress, the CYP22 protein enhances the function of splicing factors SR30 and U2AF1B by maintaining their conformational stability. Both factors contribute to plant thermotolerance by regulating the splicing of heat-responsive genes.

The small ribosomal protein RPS is crucial for mRNA decoding and the initiation of translation, whereas the large ribosomal protein RPL plays a key role in peptide chain transfer and elongation [39]. RPS and RPL collaboratively regulate rRNA cleavage during precursor rRNA processing, ensuring proper subunit assembly [40]. Darriere T. et al. found that under high-temperature stress at 37°C, the abundance of RPS in Arabidopsis decreased while that of RPL increased [41]. DnaJ proteins, as co-chaperones of HSP70, assist in the proper folding of nascent ribosomal proteins, preventing erroneous aggregation [42]. Song et al. found that the CsDnaJ gene in cucumber is significantly upregulated under high-temperature stress, indicating its important role in plant thermotolerance [43]. In our study, we identified RPS4−3 (isoform_85894), RPS6 (isoform_124694), RPL7a-1 (isoform_105123), RPL19−3 (isoform_10207), RPL27−2 (isoform_162745), and DnaJ proteins (isoform_60212, isoform_137195). This study demonstrates that DnaJ proteins (isoform_60212 and isoform_137195) are localized within the cell nucleus and show significant upregulation in response to high-temperature stress. This indicates that the DnaJ protein may reduce cell damage caused by high temperatures by stabilizing key metabolic enzymes or protecting membrane structures.

The ubiquitin-proteasome system (UPS) is one of the crucial mechanisms by which plants respond to high-temperature stress, facilitating the degradation of misfolded proteins to maintain intracellular protein homeostasis and functionality [44]. Ubiquitin-conjugating enzyme E2 is a core component of this system, regulating the ubiquitination process that determines protein degradation [45]. A. S. Caeiro et al. found that the E2 gene of Lemna minor is activated under high-temperature stress, potentially contributing to cellular homeostasis by degrading damaged proteins and regulating signaling pathways [46]. SKP1 and CDC48 collaborate in the degradation of harmful proteins under high-temperature conditions through the ubiquitin-proteasome system (UPS)-related degradation pathway, thereby jointly maintaining intracellular protein homeostasis [47]. Fan et al. found that a 6-hour heat stress treatment significantly increased the expression level of the SKP1 gene in wheat [48]. Yang et al. conducted a proteomics study revealing that high-temperature stress treatment results in the upregulation of cell differentiation regulatory protein 48 in wheat spikelets [49]. In this study, we found proteins Ubiquitin-conjugating enzyme E2 (isoform_100608), SKP1-like protein (isoform_11379), and Cell division cycle protein 48 (isoform_65297, isoform_166515). The proteins SKP1 and CDC48 are progressively upregulated with increasing temperature, whereas the expression level of Ubiquitin-conjugating enzyme E2 reaches its peak in the 35°C treatment group. These results indicate that the ubiquitin-proteasome system is activated to rapidly clear heat-damaged proteins.

HSP70 is an important member of the heat shock protein family, serving as a molecular chaperone that assists in protein folding, transport, and prevents aggregation to maintain normal cellular functions [50]. HSP70 protects cells from heat damage by binding to denatured proteins and assisting them in regaining their native conformation [51]. Usman et al. found that the upregulation of HSP70 expression in chili pepper can promote the stability of the spliceosome, thereby enhancing the plant’s tolerance to high-temperature stress [52]. HSP70 also facilitates protein refolding in conjunction with DnaJ proteins, thereby maintaining intracellular protein homeostasis and enabling a rapid response to heat treatment [53,54]. In addition, HSP70 is involved in protein degradation during endocytosis, where it binds to substrate proteins and marks them as targets for degradation [55]. Research indicates the small HSP (sHSP) is critical for plants to acquire heat tolerance, protecting cells from oxidative damage caused by high temperatures [56]. Batcho discovered that the heat shock proteins HSP70, HSP70−5, HSP15.7, and HSP17.4B in sweet potatoes were significantly upregulated under high-temperature stress [57]. This study identified the same heat shock 70 kDa protein in three pathways: spliceosome, protein processing in the endoplasmic reticulum, and endocytosis. The expression levels of HSP70 proteins (isoform_225907, isoform_230457, isoform_63193, isoform_68052, isoform_94508) are significantly upregulated across these three pathways with increasing temperature. The HSP70 (isoform_94508) protein is localized in the chloroplast, whereas the other four proteins are localized in the cytoplasm. This indicates that HSP70 proteins in *P. polyphylla* var. *yunnanensis* collaborate to maintain cellular protein homeostasis and organelle function under high-temperature stress. It also suggests that *P. polyphylla* var. *yunnanensis* may enhance heat resistance through multicellular compartmental cooperation. In the context of protein processing within the endoplasmic reticulum, we identified two small molecular heat shock proteins: HSP16.9 (isoform_176797) and HSP18.2 (isoform_101392). Both proteins are localized in the cytoplasm, and their expression levels are highest under treatment at 35°C. We hypothesize that HSP16.9 and HSP18.2 proteins may indirectly participate in the endoplasmic reticulum protein processing pathway in response to heat stress by regulating protein homeostasis.

### High temperature activates plant defense mechanisms

HY5 is a core factor in light signal transduction, participating in photomorphogenesis and responding to abiotic stress by regulating downstream gene expression [58,59]. Under high-temperature stress, casein kinase II subunit alpha-2 stabilizes HY5 through phosphorylation [60,61]. The blue light receptor cryptochrome 1 binds to SPA1, which inhibits the interaction between COP1 and SPA1, thereby promoting the accumulation of HY5 [62]. The GIGANTEA protein plays a crucial role in the photoperiod and circadian rhythms of plants and is involved in the transmission of light signals [63]. Gould et al. discovered that under high-temperature stress, GI modulates the circadian rhythm of Arabidopsis by interacting with LHY, which subsequently influences the stability of HY5 [64]. In our study, we found that the transcription factor HY5 (isoform_102627), casein kinase II subunit alpha-2 (isoform_183037), cryptochrome-1 (isoform_121679), and protein GIGANTEA (isoform_106873) were all upregulated under different temperature treatments. HY5 and CK2 were significantly upregulated with increasing temperature, suggesting that their synergistic action stabilizes HY5 through phosphorylation, thereby enhancing light signal transduction and stress responses. However, the expression level of CRY1 gradually decreased, which may indicate either the inhibition of the blue light signaling pathway or a compensatory functional response. The expression level of GI reached its peak at 35°C, indicating that it may regulate HY5 stability indirectly by modulating circadian rhythms or interacting with LHY. This suggests that GI plays a critical role in thermal responses at specific temperature thresholds, particularly at 35°C.

Glutathione metabolism plays a crucial role in alleviating oxidative stress induced by high-temperature stress in plants, by scavenging reactive oxygen species (ROS), regulating redox balance, and protecting cellular structures [65]. APX is a key enzyme in the glutathione metabolic pathway, involved in the removal of hydrogen peroxide (H_2_O_2_) and organic peroxides (ROOH) [66]. CAT is an important antioxidant enzyme that works in conjunction with APX to form a synergistic antioxidant system [67,68]. In plants such as tomato and Arabidopsis, the expression levels of ascorbate peroxidase (APX) and catalase (CAT) are significantly upregulated under high-temperature stress, indicating the crucial role of these enzymes in the adaptation of plants to elevated temperature environments [69]. APX also participates in the ascorbate–glutathione (AsA–GSH) cycle, further enhancing the antioxidant capacity of plants [70]. Glutathione S-transferase is another crucial antioxidant enzyme that plays a significant role in the synthesis and metabolism of glutathione, as well as in the clearance of reactive oxygen species (ROS) [71]. Janda et al. [72] reported that the expression of GST U18 in Brachypodium distachyon significantly increases under high-temperature stress. In this study, we found that APX (isoform_120194), GST U18 (isoform_100561, isoform_10018), NADP-ICDH (isoform_259819), and CAT1 (isoform_133789) were all significantly upregulated under high-temperature stress. This indicates that under high-temperature stress, *P. polyphylla* var. *yunnanensis* enhances its ROS scavenging ability by upregulating the expression of antioxidant enzymes such as APX, GST U18, NADP-ICDH, and CAT1, thereby activating the glutathione metabolic pathway. Cytosolic glutathione S-transferase U18 (GST U18), catalase 1 (CAT1), and NADP-dependent isocitrate dehydrogenase (NADP-ICDH) work collaboratively to maintain redox balance in the cytoplasm. In contrast, the mitochondrial-localized ascorbate peroxidase (APX) specifically safeguards the mitochondria from oxidative damage, highlighting the spatial division of antioxidant defenses.

### High temperature stress affects cell membranes

Rab7 is a small GTPase that plays a crucial role in the maturation and degradation of endosomes [73]. Mazel et al. found that the overexpression of AtRab in transgenic Arabidopsis plants resulted in accelerated endocytosis [74]. Research has shown that VPS26, VPS28, and VPS35 together form the retromer complex, which is involved in transport from endosomes to the Golgi apparatus [75]. Under high-temperature stress, VPS32 and VPS4 work together to assist plants in coping with stress by regulating the degradation process of endosomal transport [76]. In our study, we identified the proteins Rab7 (isoform_104391, isoform_175912), VPS26A (isoform_117175), VPS28 (isoform_134178), and VPS32 (isoform_143835). The VPS26A protein is subcellularly localized in the cytoplasm, while Rab7, VPS28, and VPS32 proteins are localized in the chloroplasts. Under high-temperature conditions, the expression levels of Rab7, VPS26A, and VPS28 were significantly upregulated with increasing temperature. Notably, under treatment at 35°C, the expression of VPS32 was highest compared with other treatments. This is consistent with the observation that the decline in photosynthetic indices suppresses photosynthesis, confirming the high sensitivity of chloroplasts to thermal stress. We observed that Rab7 is localized in chloroplasts and is upregulated in response to heat, suggesting its potential involvement in chloroplast protein transport or damage responses. VPS28 and VPS32 were also upregulated under high temperatures, which may be related to protein repair or degradation within chloroplasts. VPS32 reached peak expression at 35°C, suggesting the activation of key regulatory functions at specific temperature thresholds. VPS26A, localized in the cytoplasm, was concurrently upregulated, suggesting that chloroplast proteins may establish interaction networks with cytoplasmic proteins.

ADP-ribosylation factor 1 (ARF1) plays a critical role in the endocytic pathway in plants by regulating endocytosis and the internalization of plasma membrane proteins through interactions with proteins such as GNOM and VAN3 [77]. Xu et al. [78] discovered that under heat shock, the GTPase activity of ARF1 influences root hair formation by regulating the localization of ROP2 in Arabidopsis, indicating its crucial role in responding to high-temperature stress. In this study, we also identified ARF1 (isoform_102382), which is localized in the cytoplasm via the endocytic pathway. The expression level of ARF1 was significantly upregulated following high-temperature treatment, suggesting that ARF1 may play a role in the heat stress response by regulating membrane protein transport.

Autophagy is an evolutionarily conserved degradation mechanism that enables plants to cope with various environmental stresses, including high temperatures, by degrading excess or damaged cytoplasmic materials and organelles [79,80]. Research indicates that the interaction between ATG3 and ATG8 is a core component of the autophagy process [81]. ATG3 facilitates the formation and membrane tethering of autophagosomes by participating in the lipidation modification of ATG8 [82,83]. ATG8 interacts with selective autophagy receptors to participate in the degradation of specific organelles or proteins [84]. Liu et al. demonstrated that the overexpression of the ATG3 gene in maize significantly enhances the plant’s heat tolerance and improves its antioxidant capacity by upregulating the expression of other autophagy-related genes [85]. Zhou et al. reported that the expression levels of ATG8a and ATG8b in Arabidopsis significantly increased after 6 hours of exposure to high-temperature stress at 45°C, peaking after 8–10 hours [86]. In our study, we identified Autophagy-related protein 3 (isoform_116220) and Autophagy-related protein8C (isoform_11267). Protein ATG3 and ATG8C are both localized in the cytoplasm and their expression gradually increases with rising temperatures. Their cytoplasmic localization and temperature-dependent expression patterns suggest that both play a crucial role in the autophagosome formation and the clearance of damaged material induced by high temperatures.

## Conclusion

To broaden the cultivation area of *P. polyphylla* var. *yunnanensis* and to further investigate its adaptation mechanisms to high temperature environments, this study used a comparative proteomics approach and obtained 6355 quantitative proteins. A series of complex regulatory processes were found in *P. polyphylla* var. *yunnanensis* during its response to high-temperature stress. Meanwhile, several anti-stress compounds and proteins, including proline and heat shock proteins, as well as the post-translational modifications of proteins, were found to be involved in the protection and repair of functional proteins. In addition, antioxidant enzymes play an important role in removing excess ROS to reduce oxidative stress. These findings contribute to further understanding of the mechanism of action of *P. polyphylla* var. *yunnanensis* in response to high temperature stress at the protein level.

It should be noted that in this study, temperature treatments were applied as discrete, sustained exposures at constant temperatures rather than as a cumulative gradient. While this design allowed comparison of responses at specific temperature thresholds, it does not separate acute responses from chronic adaptation or cumulative damage. Therefore, the observed changes represent integrated outcomes of both stress intensity and exposure duration. Future studies incorporating time course sampling or gradual temperature increases will be needed to further dissect the temporal dynamics of heat stress responses in this species.

## Supporting information

S1 Table Primer sequences used for RT-qPCR validation. (XLSX)
